# Patients’ perspectives on the experience of absconding from a psychiatric hospital: a qualitative study

**DOI:** 10.1186/s12888-021-03382-0

**Published:** 2021-07-26

**Authors:** Mark Mohan Kaggwa, Anita Acai, Godfrey Zari Rukundo, Sheila Harms, Scholastic Ashaba

**Affiliations:** 1 Department of Psychiatry, Faculty of Medicine, Mbarara University of Science and Technology, P. O. Box 1410, Mbarara, Uganda; 2 Department of Psychiatry and Behavioural Neurosciences, McMaster University, Hamilton, Canada

**Keywords:** Mental illness, Psychiatric hospitals, Stigma, Caregivers, Absconding, Infantilization, Loneliness, Mental health system, Uganda, Escape

## Abstract

**Background:**

Absconding (i.e., escaping) is common among patients with mental illness admitted to psychiatric hospitals. Patients use various strategies to make absconding successful due to the experiences faced during admission. We conducted a study to identify patients’ perspectives on the experience of absconding from the psychiatry facility.

**Methods:**

We conducted 10 in-depth interviews with patients with a history of absconding from the hospital who were accessing care at the Mbarara Regional Referral Hospital in Mbarara city Uganda. Interviews were audio-recorded, translated when required, transcribed into English, and analyzed thematically to identify relevant themes.

**Results:**

Participants ranged in age from 18 to 55 and the majority (*n* = 9) were male. Most had absconded at least twice from a psychiatric facility. We identified different experiences that influenced patients’ engagement in absconding from the psychiatry hospital ward. These included: (1) stigma, (2) experiences with caregivers: mixed emotions, (3) poor resources and services, and (4) the influence of mental illness symptoms. The loneliness of stigma, negative emotions associated with the loss of important roles given the nature and framework of caregiving on the psychiatric ward, as well as the stress of limited resources were a salient part of the patient experience as it relates to absconding.

**Conclusion:**

Our findings indicate that absconding is a symptom of a larger problem with a mental health system that perpetuates stigma in its design, isolates patients and makes them feel lonely, and forces patients to rely on caregivers who infantilize them and take away all their freedom in a facility with no basic services. For many patients, this makes absconding the only option. Within such a system, all stakeholders (policymakers, health-care providers, caregivers, and patients) should be involved in rethinking how psychiatric facilities should be operated to make the journey of patient recovery more positive.

**Supplementary Information:**

The online version contains supplementary material available at 10.1186/s12888-021-03382-0.

## Introduction

When patients leave the psychiatry hospital without permission from healthcare workers, it is considered absconding (also known as escaping or eloping) [1, 2]. In high-income countries, 1 to 15 patients per hospital abscond per year [1]. The global annual absconding rate for psychiatry patients ranges between 2.5 to 34%. The rates are particularly high in Africa. For example, the absconding rates for psychiatric patients in South Africa is at 7.83 [3], whereas the rates are even higher in Uganda because about 10 to 50 patients are estimated to abscond every month from the National Mental Health Referral hospital, the most secure mental facility in the country [4, 5].

Despite the different rates and locations, knowledge about various factors associated with this phenomenon as well as the causes have remained similar over the years [2, 3, 6–13]. These include younger age, male sex, longer length of hospital stay, personality disorders, substance abuse disorders, patients who have been referred to the psychiatric hospital by police, those with employment problems, and influence of psychiatric symptoms [2, 3, 6–13].

Absconding from psychiatric hospitals is associated with slower recovery and prolonged hospitalization due to the interruption in treatment [7, 14]. Despite the health challenges associated with absconding, the literature would suggest that patients’ experience at the facilities strongly affect their decisions to abscond. Patient perspectives from developed countries have reported lack of social support from friends and family, a lack of freedom, a feeling of being confined, boredom, poor doctor-patient relationships, problems with medications, disturbance from other patients, and poor quality of food within the hospital, which makes them dislike the hospital environment, thereby leading to absconding [1, 3, 11, 15]. Some have experienced fear in response to feeling as though their safety is threatened, harassment, being overtly threatened by other patients, or having had their property stolen [2, 12, 13]. These results have largely been obtained by using quantitative methods, although some qualitative work has looked at understanding causal factors for absconding based on informants involved in patients care and former absconders experiences [16–19].

Despite the large cultural differences and employed methods in patient care across the globe, such as direct involvement of caregivers in patient care; to the best of our knowledge, there are no qualitative studies in Africa that have explored patients’ emotional experiences of psychiatric hospitalization and its relationship to absconding. This study aimed to qualitatively explore patients’ perspectives and emotional experiences related to the phenomenon of absconding from a psychiatric hospital in Uganda, as well as to understand what it means for patients to experience life in a psychiatric ward.

## Methods

### Study design

This was a qualitative descriptive study [20] involving in-depth interviews with patients with mental illness who had previously absconded from the Mbarara Regional Referral Hospital (MRRH) Psychiatry Unit. This study was reported in accordance with the COnsolidated criteria for REporting Qualitative research (COREQ) checklist [21].

### Study setting

This study was conducted in the Psychiatry Unit at MRRH, a hospital located in Mbarara City in southwestern Uganda. MRRH is located 270 km from Kampala, the capital city of Uganda. In MRRH’s Psychiatry Unit, psychiatric care is unique in that psychiatry patients have their caregivers directly involved in their care and all admitted patients are typically accompanied by a caregiver. The majority of patients who seek care at MRRH live in rural areas outside Mbarara Town and the surrounding districts [22].

### Participants

Participants in this study were patients with mental illness in a remission phase who were attending the outpatient mental health clinic at MRRH. We purposively selected and included patients with prior history of absconding from the psychiatry unit and stable in their course of mental illness. Eligible participants were identified by a nurse on the Psychiatry Unit from patients attending the outpatient clinic. They were given an overview of the study and, upon consent, an appointment for data collection was made. A reminder message was sent before the set date for the interview.

Participants ranged between 18 and 55 years of age. Nine participants were male, and one was female. Nine of the ten participants were unemployed and only one had attained a tertiary level of education. The median years with mental illness was eight and a median number of times that a patient had absconded was two. Five participants were single, three were separated or divorced, and two were married. The most common diagnosis among the participants was bipolar affective disorder (*n =* 6), while two patients were being managed for schizophrenia, and two were being managed for substance use disorders. The median number of years with mental illness was eight, and the median number of admissions was four. We excluded participants who were physically and psychologically too sick to participate in the study or give us reliable information.

### Data collection

Interviews were conducted by a research assistant who was trained in qualitative data collection methods. Interviews took place between September 8th, 2020 and September 30th, 2020. The interviews were scheduled based on participants’ availability and lasted between 30 to 60 min (average: 40 min). Interviews were conducted in participants’ preferred language. The interview guide was generated through reading literature relevant to absconding of patients from psychiatric hospitals and with input from mental health experts in the region. Sample questions from the semi-structured interview guide (Supplementary Material 1) included, “Tell me about what it is like to a patient with mental illness at the Mbarara Regional Referral Hospital?” and “Tell me about the time you escaped from the psychiatry ward.”

### Analysis

Interviews were audio recorded, translated when required, and transcribed into English. Only one of the participants was fluent in English and could read the transcribed interview to confirm accuracy of the translated and transcribed interview. The summaries of the interviews were read out to the participants to check if the interpretation of the information was rhyming with their experiences. Analysis occurred concurrently with data collection by the research team (MMK, SA, GZR, SH, AA) using thematic analysis [23] to understand the patient experience and reasons for absconding from the psychiatry hospital during their admission. The specific steps involved in thematic analysis include familiarizing oneself with the data, generating initial codes, searching for themes, reviewing themes, defining and naming themes, and producing the report [23]. After reading three transcripts each, the research team developed a codebook to guide analysis of the qualitative data through line-by-line coding of all the transcripts. After coding five interviews in duplicate, AA and MMK coded the remaining transcripts independently. Throughout the analytic process, the research team held regular meetings to harmonize the identified themes and to discuss their interpretations of the data, including reviewing the field memos made during data collection, and developing themes from related codes. The team reflected a collaborative effort between researchers from Mbarara University in Uganda and McMaster University in Canada, led by SA and SH, respectively. The team comprised MMK (clinical psychiatry resident), SA (PhD, experienced qualitative researcher, senior lecturer, and clinical psychiatrist), and GZR (PhD, experienced qualitative researcher, senior lecturer, and clinical psychiatrist) from Uganda and SH (experienced qualitative researcher, associate professor, and clinical psychiatrist) and AA (experienced qualitative researcher, assistant professor, and PhD-trained education scientist) from Canada. The diversity of the research team helped make more explicit researcher characteristics that influenced interpretations of the data. After analysis, member checking [24] was done with one participant. Data were collected until no new ideas were articulated by the participants.

### Ethics

The study received ethical approval from research ethics committee of Mbarara University of Science and Technology (# 17/06–20). Permission to collect data from participants was granted by the director of MRRH. All participants provided voluntary informed, written consent at study enrollment.

## Results

### Experiences influencing absconding from the psychiatry hospital

We identified different experiences that influenced patients’ engagement in absconding from the psychiatry hospital ward from 10 interviews. Using thematic analysis, we summarized the findings into four main themes including: (1) stigma, (2) experiences with caregivers: mixed emotions, (3) poor hospital resources and services, and (4) the influence of mental illness symptoms. We describe each of these themes below.

#### Theme 1: stigma

Patients did not want to be seen in a psychiatry hospital. They described hatred towards being admitted in a psychiatry ward as it reportedly made them feel inferior to others. The experience of hospitalization also led to confusion and distressing beliefs about how they developed a mental illness. One participant illustrated this feeling in the following quote:

*“I feel stigmatized. I would wonder what I am doing among patients with mental illness … I am not ill, a full man like me who studied engineering and is supposed to be at [work], so from that I would look for a way out and I absconded.”* (Participant 10)

The facilities were felt to be designed in a way that promoted stigma. Some participants described dreading the experience of being put into seclusion rooms, describing it as a horrific experience. Those who were put in seclusion lived in fear of being secluded again, influencing their thinking and feeling as though they needed to abscond from the hospital. Many patients described the experience of being in the seclusion room as a dehumanizing encounter. A patient described his fears associated with the seclusion room as follows:

*“The seclusion room made me so hungry to the extent that I felt like dying … I could not hold it anymore and I had to eat a whole T-shirt, the one I was putting on. It is a terrible place, and I cannot wish any person to be isolated there. It is just a cool dark room … but the problem is that there is no food and no freedom. You cannot see or talk to any person; somehow, it makes you get scared.”* (Participant 7)

Some participants compared being in the psychiatry hospital to being in prison, where their movements were restricted and they lacked the freedom to do what they wanted. This environment was described as leading to patients feeling as though they were being contained not because of their illness, but because they were a menace to the community, deserving of punishment. Thus, for some participants, absconding was a way of regaining freedom, dignity, and independence:

*“We live in a situation whereby all the time you are under watch and control … you feel as if you are useless and that you cannot do anything for yourself.”* (Participant 8)

#### Theme 2: experiences with caregivers: mixed emotions

Some patients reported that their caregivers on the psychiatry ward could, at times, treat them like infants whose everyday activities needed to be controlled. Patients noted that expectations of caregivers, which included remaining in the hospital at all times to provide for patients, was not always experienced as supportive or comforting. For example, caregivers would routinely take on tasks which included washing clothes for patients, purchasing food, getting medications, and bathing them. Tasks that patients could normally do independently, were removed from them and taken over by caregivers, which was experienced as minimizing for them and also resulted in profound boredom. This unintended infantilizing exacerbated feelings of being of no use:

*“Sometime [s] you feel you want to go to church and there is no way because people around you cannot allow you to move. In case you want to buy anything for yourself, there is no time or space for you to do so.”* (Participant 8)

*“Sometimes they try to stop me especially my mother … she does not want me to move around. But if I get the chance, I do the things I want like buying anything I want from the shops.”* (Participant 7)

*“But all in all, this condition is not good … . there is nothing to do.”* (Participant 5)

Other study participants were acutely aware of the burden that was associated with caregiving, leaving patients feeling guilty and burdened themselves as they watched their own caregivers become overwhelmed and distressed. Patients spoke about hearing their caregivers express their own intense emotions, resulting in an added negative experience for them that was associated with wanting to escape. Patients were aware, in particular, of the monetary costs associated with caring for them, leading to a kind of desperation for both caregiver and patient which was thought to be remedied through absconding:

*“The only challenge is that the caregivers are really bothered a lot: looking for food to feed their patients outside the facility, they also actually do not have enough money with them. This reason makes caregivers request for an early discharge or abscond with their patients, even when the patient is not yet cured properly.”* (Participant 4)

Another patient described the loneliness that was associated with having caregivers around but not being attended to by them, thereby worsening their experience:

*“The reason why patients with mental illness abscond is because caregivers do not take care of them. If their caregivers are always by their side, then they wouldn’t think of absconding. But when they are alone, they get the idea of absconding.”* (Participant 2)

Other study participants talked about noticing how caregivers managed the stress of dealing with sick patients through physical violence and abuse. This kind of treatment directed at patients was noted and experienced as highly distressing, as well as being associated with a kind of helplessness. These feelings of distress often culminated in a double need to escape, which included escape from hospital as well as escape from abusive caregivers:

*“Caregivers are different. There are caregivers who, when their patients annoy them, they react by hitting them, but they do it with good intentions to scare you from doing wrong things.* (Participant 5)

*“In most cases, what is so challenging and annoying, is when you are in such a condition (having mental illness) and you find that people (caregivers) are just abusing you … you also feel uncomfortable being around such people because, at the end of the day, we do not ask to be in such a situation.”* (Participant 8)

#### Theme 3: poor hospital services

Poor hospital facilities also appeared to be associated with absconding. The living conditions were described as poor with overcrowding, poor staffing, limited to no security, as well as lacking in basic services such as places for cooking and engaging in self-care.

##### Unpleasant living conditions in the psychiatry hospital

Some participants mentioned that the living conditions at the hospital were unpleasant. Substandard levels of cleanliness across all living spaces, the use of seclusion rooms, the absence of methods like games or sports to engage patients, a lack of basic services such as providing food, and overcrowding were all factors that were associated with absconding. This is illustrated by the following quotes:

*“The toilets at this hospital are always dirty so we find it hard to use them”* (Participant 2)

*“ … you feel uncomfortable with the smell of the medicines that are all over the hospital all the time. It is disgusting.”* (Participant 1)

##### Porous facilities

Participants mentioned that the psychiatry hospital was not well secured, as identified by an open gate and complacent or absent security personnel. In addition, patients found it hard to differentiate between other patients and their caregivers since they did not have identification cards or uniforms. This made it easy for patients to leave the hospital with no one suspecting:

*“I did not use any trick to abscond. I just passed through the gate and no one stopped [me] because I had no luggage. I passed by the gate like I was just going outside but I never came back.”* (Participant 2)

In addition, participants mentioned that the psychiatry hospital lacked basic security measures of maintaining patients within its boundaries, as illustrated by this participant:

*“Since this psychiatric hospital has no security lights and is not fenced with electric wires, you can easily jump over the fence and escape. And there is no security at the gate so it would be easy to just pass and abscond.”* (Participant 3)

##### Lack of basic services

A lack of basic services provided to patients by the hospital, most notably food, was another reason given for absconding. Participants noted that their caregivers could not always afford to buy food for them, leading patients to spend days without consuming anything but their medications. One participant described how a lack of food forced him to abscond from the hospital:

*“I was left here at the hospital alone, so I became lonely. It is always better for patients with mental illness to stay with their caregivers [because] they need food to be available, but I had none of that. It was 4:00pm and I hadn’t eaten anything, so I escaped and decided to go home so that I could maybe get [something] to eat.”* (Participant 2)

*“The issue of feeding remains a challenge … In most cases, we do not feed very well, not like we feed when we are at home and, in addition to that, we cannot have access to freedom as human beings just like when we are out there with friends and family.”* (Participant 5)

Overcrowding in the psychiatry hospitals was another reason for a perceived need to abscond. This phenomenon also affected caregivers such that absconding was a preferable solution to the conditions that needed to be endured at the hospital.

*“ … the living conditions of the caregivers is also not good. Sometimes the patients together with the caregivers are so many, that the space provided is so limited for both, this may prompt the caregivers also to assist the patients in escaping from the facility because of the poor conditions.”* (Participant 8)

##### Loneliness and seclusion

The intolerable environment of the psychiatry hospital was perceived to be worsened by the severely damaged social connectedness that was a result of the hospital experience. Many patients were seeking social engagement and relationships but were often unable to find this. This was clearly illustrated in the following memo describing the patient experience:

*"This patient is describing the loneliness of being on a journey of recovery. They are not at their worst, but also not at their best. They are invisible to others; they can escape without being seen or heard. They want to wear a uniform—something that makes them identifiable and distinguishable from others. Like food, social connection is a basic need. The attempt to escape loneliness is futile, as it is always there. Even when the patient is at home, they say they are waiting for something.*

Moreover, according to some participants, being in the hospital was extremely boring due to the lack of engagement in meaningful activities. Thus, patients getting tired of their daily could also motivate their desire to abscond:

*“I was exhausted from the hospital because all I would do is sleep all day and swallow medicine. I knew that if I went to the village, I would at least go to the pitch and watch a football match.”* (Participant 4)

##### Financial challenges

Participants noted that most patients with mental illness are not employed and face financial challenges such that they depend on their caregivers to purchase the items that are required for basic existence in the hospital. This could become costly for caregivers, who had to ensure that patients got three meals a day; yet, the majority of caregivers were not financially stable themselves, leading patients to go without their basic needs being met. A participant illustrated how financial challenges forced them to abscond from the hospital:

*“Most patients abscond due to lack of financial support. … I was given money just in case I wanted to buy a Rolex [snack], but just because I wanted us to save money and economize, I would tell them, ‘Let's go home.’ But there are patients that do not have money to afford buying food all the time, so this causes them to escape.”* (Participant 4)

#### Theme 4: the influence of mental illness symptoms

Participants reported that symptoms of mental illness were, in part, responsible for patients absconding from the hospital. For example, the presence of psychotic symptoms during admission, particularly commanding auditory hallucinations, instructed patients to leave the hospital. Many felt compelled to obey these voices. Others mentioned that persecutory delusions made them scared of everyone around them, leading them to feel unsafe in the hospital. Hence, they absconded to find safety away from the hospital. Participants’ experiences are described below.

*“When you are escaping it’s because you become scared that people around you are going to kill you, so you have that sense of fear, your minds tells you, ‘Why don’t you get out of this place because these people will kill you,’ so you get motivated and do whatever you can to escape.”* (Participant 3)

*“Once, I escaped because I heard a voice from God telling me to leave the hospital and go to church. The message was so strong that I had to do everything possible to be in church, so I absconded. When I reached church, the messages stopped.”* (Participant 8)

Other symptoms associated with some types of mental illness were reported to be responsible for patients’ absconding behavior. For example, some patients had a high libido and described this as being one of the reasons why they absconded from the hospital. The need for sex motivated them to leave the hospital at any cost, as illustrated by the following quote:

*“By the way, when I get attacked [by mental illness], I always have a mindset of knowing where my home is, so I think about going back home and I think about having sexual relations with my wife, so I feel like I am missing out in the process of delaying here while receiving treatment, so this motivates me to abscond … ”* (Participant 3)

Other participants reported that during admissions, they were occupied with thoughts of leaving the hospital and described a kind of desperation, admitting to a willingness to do anything to make sure they could remove themselves from the restraints of the hospital setting. In the following quote, a participant illustrated how patients could become deceitful in order to abscond and stop these thoughts:

*“First, I would develop thoughts of absconding and going back home … so, I would simply lie to my mother or any other person taking care of me that I am going out to use the toilets and I just disappear from there … and go home.”* (Participant 3)

## Discussion

Our study explored the emotional experiences associated with absconding from a psychiatric ward at a Ugandan hospital. The four study themes (stigma, experiences with caregivers: mixed emotions, poor resources and services, and the influence of mental illness symptoms) can be broadly grouped into three experience categories including the loneliness of stigma, negative emotions associated with the loss of important roles given the nature and framework of caregiving on the psychiatric ward, as well as the stress of limited resources as a salient part of the patient experience as it relates to absconding. As suggested by Brumbles et al. [25], stigma is a motivator for absconding because patients reported feeling inferior as a result of having a mental illness. Stigma associated with mental illness in Uganda is high [26]. Despite a patient being severely mentally ill, many patients in the current study did not want to associate with mental illness setting a scenario where absconding was perceived by the patient as an effective solution to the problem of stigma. Patients in the study described not being engaged in the psychiatric ward that eliminated their ability to engage in meaningful roles and left them bored. This loss of social status is another way in which stigma was experienced by patients. For example, in the Ugandan context, a man who is prevented from doing work associated with providing for his family is equivalent to a loss of purposeful identity. Therefore, the experience of doing nothing on the ward was also deeply distressing, which led to absconding, despite having caregivers (i.e., family members) present.

In addition, the physical structure of the facility was described as promoting stigma through the presence of the seclusion room. Simply being aware of a space that could be used to single out and separate patients from others was perceived as a threat. Patients articulated a kind of assault on their dignity when put in seclusion by healthcare workers and/or their caregivers. Although these reports are congruent with other psychiatric patient perspectives in Uganda where being put into seclusion by caregivers was described as highly stigmatizing [27], other experiences further perpetuated the stigma experienced by patients in our study. Patients reported being fearful and vulnerable about the possibility of being put into seclusion by anyone on the ward, including by other psychiatric patients, caregivers of other patients, the patient’s own caregiver, as well as by the healthcare workers. This scenario led to a kind of experience that felt unpredictable and unsafe because of the status of being a patient. The fears and horrors of being forced into seclusion have been described in previous studies [28, 29] as an issue of life or death. The patient experience here turns from one of unpredictability to feeling as though the hospital is associated with serious danger, the potential for assault, as well as a threat to one’s life. These kinds of traumatic emotions are understandably linked to dramatic actions described by one patient in this study who reported eating his own T-shirt while in seclusion due to hunger. Absconding for this patient is understood as the least traumatic of his encounters while in hospital. For the patients in our study, a place of ‘healing’ became a place of incarceration such that there is double stigma and makes having mental illness feel like a crime.

In most health facilities in Uganda, there is a shortage of staff, making it necessary for caregivers to be involved in patient care [27, 30]. Caregivers of patients with mental illness, who are often their own family members, have a high burden of care characterized by multiple responsibilities, which affects their ability to provide adequate care for their patients [31]. The burden increases with the severity of the patient’s illness [32] but also leads to the stripping of almost all patient independence. Caregiving process is described as a process of caring for a patient with mental illness to be akin to caring for a small child (infantilization). Care involves feeding, bathing, and constantly monitoring the patient to ensure that they do not cause harm to themselves or others. Despite being in the presence of constant care [33], patients in our study still described negative feelings such as loneliness, boredom, and even resentment as a result of having their dignity stripped away. As a result, absconding became a way for patients to return to some level of independence and control over their own lives.

In this study, the health facilities were found to have poor services that made patients have a negative experience during admission, hence motivating their desire to elope. The living conditions were poor, with questionable security, overcrowding, poor staffing, and a lack of basic services, such as cooking facilities for patients. These poor services have been identified by many researchers, and are mainly attributed to poor mental health policies and laws [27, 33]. Uganda had taken over 50 years to update its mental health treatment policy, and the use of seclusion rooms, urgency orders, and derogatory language such as “person of unsound mind” or “idiots” to describe patients with a mental illness, continues to perpetuate stigma around the topic of mental health [33, 34].. This is exacerbated by financial constraints, as the total amount of money allocated to mental healthcare in Uganda is less than 1% of the health budget [33]. This is not enough to improve services offered by psychiatric facilities and perpetuates the issue of absconding. In South Africa, absconding was nearly cut in half after a change in the country’s the Mental Healthcare Act [3]. Government intervention is urgently needed to help address the issue of absconding in Uganda.

### Limitations and future direction

The study findings should be interpreted in light of some limitations. In qualitative methodology, member checking is one way of ensuring the trustworthiness of the data. While patients read their own transcripts, member checking was not completed by having patients read over the full set of themes. Although this study was intended to be descriptive, there is an inherent methodological limitation in that the prioritization of factors related to absconding could not be determined. Future studies could explore this in more detail. This was a single institution study, which may be a shortcoming in generalizing suggestions made by the authors directed at government and policy to assist with improving patient experiences.

## Conclusions

Our findings indicate that absconding is a symptom of a larger problem with a mental health system that perpetuates stigma in its design, isolates patients and makes them feel lonely, and forces patients to rely on caregivers who infantilize them and take away all their freedom in a facility with no basic services. For many patients, this makes absconding the only option. Within such a system, all stakeholders (policymakers, health-care providers, caregivers, and patients) should be involved in rethinking how psychiatric facilities should be operated to make the journey of patient recovery more positive.

## Supplementary Information

**Additional file 1.** Interview guide.

## Data Availability

The datasets used and/or analysed during the current study are available from the corresponding author on reasonable request.

## Abbreviation

MRRH: Mbarara Regional Referral Hospital
