# Supplementary material for: Patients’ perspectives on the experience of absconding from a psychiatric hospital: a qualitative study
Source: BMC Psychiatry. 2021 Jul 26;21:371. doi: 10.1186/s12888-021-03382-0 (PMC8311958; doi:10.1186/s12888-021-03382-0)
Supplement: Supplementary file 1 — Additional file 1. Interview guide. [file 12888_2021_3382_MOESM1_ESM.docx]

**Supplementary material I: Interview guide**

Hello. My name is _________________________ and I am part of the team doing a study looking at causes, tricks, and solutions to escape of patients at Mbarara Regional Referral Hospital. Just like you, many patients end up escaping from the hospital due to various reasons, several tricks are used to escape. I want to hear about your experiences during the period you escaped. Our goal is to use this information to help improve patient care and reduce escape of patients from the hospital.

As we discussed, our interview will be audio recorded. No other person apart from the research team of the study will be able to listen to the recording, and it will be deleted when we have finished the study.

We have just reviewed the consent form, which describes the study in detail and gives us permission to speak with you.

Do you have any questions before we begin the interview?

| 1. Tell me about what it is like to be a patient with mental illness admitted at Mbarara Regional Referral Hospital | Explore  · What they were managed for?  · How long have you been in management for mental illness?  · How many times have you been admitted?  · How do the health workers behave towards patients?  · Have you ever escaped? |
| --- | --- |
| 2. Tell me about the time you escaped from the psychiatry ward | Explore  · What made you escape?  · How did you escape (tricks)?  · Did you feel stigmatized because of being admitted?  · Was your caregiver involved in your escape?  · Did the health workers contribute to your escape?  · Did you have financial difficulties  · Was it lack of food?  · Responsibilities at home and community/work |
| 3. How many ways would you have escaped from the hospital |  |
| 4. How do you feel about the psychiatry ward? |  |
| 5. Do you believe that you have mental illness, and admitting you was the right decision | Explore  · Do drugs give you side effects?  · Is this a spiritual illness, not medical?  · Are the doctors wrong? |
| 6. What should we do to reduce the act of escaping from the psychiatry ward |  |
